# Influence of Anatomical Parameters on the Dimensions of the Subantral Space and Sinus Mucosa Thickening after Sinus Floor Elevation. A Retrospective Cone Beam Computed Tomography Study

**DOI:** 10.3390/dj9070076

**Published:** 2021-06-24

**Authors:** Yuki Omori, Yasushi Nakajima, Hideki Imai, Daichi Yonezawa, Mauro Ferri, Karol Alí Apaza Alccayhuaman, Daniele Botticelli

**Affiliations:** 1 Department of Oral Implantology, Osaka Dental University, Osaka 573-1144, Japan; info@omori-dent.com (Y.O.); y.nakajima@me.com (Y.N.); imaihideki@gmail.com (H.I.); 2 Department of Applied Prosthodontics, Graduate School of Biomedical Sciences, Nagasaki University, Nagasaki 852-8102, Japan; 3 ARDEC Foundation, 130001 Cartagena de Indias, Colombia; medicina2000ctg@hotmail.com; 4 Department of Oral Biology, Medical University of Vienna, 1090 Vienna, Austria; caroline7_k@hotmail.com; 5 ARDEC Academy, 47923 Rimini, Italy; daniele.botticelli@gmail.com

**Keywords:** antrostomy size, biomaterial, cone-beam tomography, maxillary sinus, palatal–nasal recess, posterior superior alveolar artery, sinus augmentation, sinus height, sinus septa, xenograft

## Abstract

Background: Various anatomical parameters might influence the surgical approach for maxillary sinus floor elevation. The objective of the present study was to retrospectively evaluate the influence of anatomical parameters on the dimensions of the subantral space and of the sinus mucosa thickening after sinus floor elevation. Material and Methods: Seventy-eight maxillary sinuses in sixty-five patients were evaluated on cone beam computed tomographies taken before surgery and after one week (t1w) and nine months (t9m). Several parameters such as the distance XF between an axis parallel to the base of the nose (X-axes) and the sinus floor (F) were correlated with the height gain (IF) at t1w and t9m and the post-surgical edema. Results: A weak significant positive correlation was observed between height gain vs. sinus height of interest (XF), the balcony, and the sinus floor angle. The post-surgical edema was influenced by the initial mucosa thickness and the xenograft used. Conclusions: Various parameters might affect height gain and sinus mucosa thickening after sinus floor elevation. The height of interest, the balcony, and the sinus floor angle showed significant correlations with height gain. The initial thickness of the mucosa and the biomaterial used influenced the post-surgical edema.

## 1. Introduction

The posterior region of the maxilla often requires the augmentation of the sinus floor with the aim to obtain sufficient bone volume for implant insertion. Sinus floor elevation by applying a lateral approach is a well-known technique that has been shown to have a high rate of success [1]. Nevertheless, anatomical conditions might generate technical problems so that an accurate examination of the region on a cone beam computed tomography (CBCT) prior to the surgery is strongly recommended [2,3]. This radiographic analysis allows the clinician to evaluate the presence of lesions within the sinus [4,5] or the presence and conformation of septa when present [6,7,8]. Both these conditions increase the difficulty of the surgery. However, the radiological analysis of the anatomy also allows the assessment of various parameters such as the height of the residual bone crest, the angles of the sinus floor and the palato-nasal recess, the width of the lateral bone wall, and the thickness of the sinusal mucosa. Suggestions on what to measure and how to take measurements on CBCT images have been provided in several studies [2,9,10,11,12]. The use of the base of the nasal floor as a reference plane was adopted for both bi-dimensional [10,11,13,14] and 3-dimensional [15] evaluations. That axis delimits the lower region of the sinus where the implant will be inserted. Obviously, when the axis is too close to the sinus floor, part of the sinus above the axis will also be occupied by biomaterial and by the apical segment of the implant. Nevertheless, the use of such a reference plane has been shown to be helpful for radiographic measurements, and allows one to obtain repeatability of the data, making possible comparisons with data from CBCTs of other patients or from the same patient. The dimensional changes of the elevated space over time can be easily assessed [10,11,12,13,14]. Several studies have been published reporting the dimensions of the elevated space after sinus floor elevation and the dimensional modifications over time [10,11,13,14,15,16,17,18,19]. Other anatomical and surgical parameters have been analyzed in various reports. The edema underneath the sinus mucosa that yields the increase in its dimensions after sinus floor elevation as evaluated on CBCTs has been reported in various clinical studies [20,21,22]. The evaluation of the palato-nasal recess (PNR) has been assessed in RCTs [13,14] and retrospective studies [2,23]. In a retrospective study, it was shown that PNR <90% could increase the risk of perforation of the sinus mucosa during the surgical procedure [23]. The influence on sinus mucosa perforation of the amplitude of the angle between the lateral and medial walls of the sinus (sinus floor angle; SFA) has been evaluated in a clinical study [24]. A higher risk of perforation was observed with SFA < 30°. The height of the bone crest was associated with a risk of sinus mucosa perforation rate even though different outcomes were reported. In most studies, it was concluded that the lower was the height, the higher the risk of perforation [25]. However, a study failed to show differences [26].

The association between the lateral wall thickness and perforations was also evaluated in clinical studies [26,27]. It was shown that the thicker the bone wall, the higher the risk of perforations. The influence on the height of the elevated space of the antrostomy dimensions and its position in relation to the sinus floor has also been discussed in clinical studies [10,11,16]. While no differences were found regarding the antrostomy dimensions [11,16], the more cranial the antrostomy, the greater the augmentation height obtained [10].

However, the correlations between the various anatomical factors and the elevated space dimensions and mucosa increased thickness have not been extensively evaluated yet. Hence, the objective of the present study was to retrospectively evaluate the influence of anatomical parameters on the dimensions of the subantral space and of the sinus mucosa thickening after sinus floor elevation.

The clinical relevance of the present retrospective study was to focus the attention of the clinicians on the anatomical parameters and surgical approach to be applied to obtain the expected bone height for implant insertion and to reduce the post-surgical sub-mucosa edema that might trigger complications subsequent to the obstruction of the ostium and infundibulum [28,29].

## 2. Materials and Methods

### 2.1. Study Population

Sixty-five patients, forty-one females (mean age 53.3 ± 8.6 years), and 24 males (mean age 58.0 ± 10.4 years), were included in this retrospective study. Patients that consecutively underwent maxillary sinus augmentation at the University Corporation Rafael Núñez of Cartagena de Indias, Colombia, from August 2015 to March 2018 were analyzed using CBCTs. All patients included in the present additional data analysis participated in randomized controlled trials that received the following approvals by the Ethical Committee of the Corporación Universitária Rafael Núñez, Cartagena de Indias, Colombia (protocol #01-2015, 19 May 2015; protocol #02-2015, 19 May 2015; protocol #03-2015; 4 December 2015). To be included in the previous RCTs, the patients had to fulfill the following inclusion criteria: (i) presence of an edentulous region in the posterior region of the maxilla; (ii) height of the bone crest ≤4 mm; (iii) need of an oral restoration with fix prosthesis supported by implants; (iv) ≥21 years of age; (v) good general health; (vi) no contraindications for oral surgical procedures; and (vii) not being pregnant. The patients were not admitted to the study if they: (i) presented a systemic disorder; (ii) had chemotherapy or radiotherapy; (iii) were smokers >10 cigarettes per day; (iv) had an acute or a chronic sinusitis; and (v) had previous bone augmentation procedures in the region of interest.

In the present study, all data were collected and the unpublished correlations among various parameters were retrospectively evaluated. Only the patients that had all the CBCTs (cone beam computed tomographies) taken before the maxillary sinus augmentation (t0) and after one week (1w) and nine months (9m) were included. The lack of radiographic and clinical data in the three stages of the examination was considered an exclusion criteria.

### 2.2. Surgical Procedures

The surgical procedures have been illustrated in articles already published [10,11,12,13,14] and a short description is included in the present paper. After the exposure of the lateral wall of the maxillary sinus, an antrostomy was prepared by grinding the bone with a round diamond insert (SFS 109 029), Komet-Brasseler-GmbH, Lemgo, Germany), mounted on a sonic-air surgical instrument (Sonosurgery^®^ TKD, Calenzano, FI, Italy). The sinus mucosa was subsequently carefully elevated and the space was filled with xenografts (Gen-Os, OsteoBiol, Tecnoss, Giaveno, Italy or Cerabone, Botiss Biomaterials GmbH, Zossen, Germany). A collagen membrane to protect the antrostomy was placed in most cases and the wound was sutured. After six months, the healing of the xenografts used was considered sufficient [30,31] to insert mini-implants in all patients for histological analysis. After a further three months, the mini-implants were harvested for histological evaluation, the results of which are reported elsewhere, and the final implant was inserted in the same position.

### 2.3. Biomaterials Used

Gen-Os (porcine xenograft) was composed of granules, 250–1000 µm in dimension, of porcine bone treated at a low temperature of up to 130 °C to eliminate the pathogens and allow the preservation of structure and composition of both collagen and hydroxyapatite.

Cerabone (bovine xenograft) was composed of granules, 1000–2000 µm in dimension, of bovine cancellous bone treated at a high-temperature process (>1200 °C).

OsteoBiol Evolution membrane (OsteoBiol, Tecnoss, Giaveno, Italy) made of porcine heterologous mesenchymal tissue was used to protect the antrostomy of sinus elevated with Gen-Os.

Collprotect membrane (Botiss Biomaterials) made of porcine collagen from dermis was used to protect the antrostomy of sinus elevated with Cerabone.

### 2.4. CBCT Imaging Analyses

All CBCTs were taken in the same radiological center using a 3D Accuitomo 170 Tomograph (J Morita Corporation, Kyoto, Japan). The CBCT images were recorded at 80 kV and 8 mA, FOV 77.125; 77.125; 74.000. The 3D reconstruction was performed with slices at an interval of 1.0 mm with a basic voxel size of 0.125 mm.

**Figure 1:**
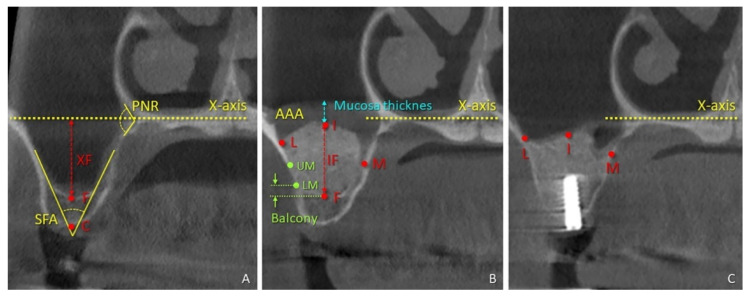
References for the measurements performed on CBCTs (**A**) before surgery, (**B**) after 1 week (**B**), and (**C**) 9 months. *X*-axis, plane placed at the base of the nasal floor; PNR, palate-nasal recess; F, sinus floor; C, top of the bone crest; XF, distance between *X*-axis and F; SFA, sinus floor angle; UM, upper margin of the antrostomy; LM, lower margin of the antrostomy; L, I, M, the uppermost extension of the hard tissue within the elevated space at the lateral, intermediate and medial aspects, respectively; AAA, alveolar-antral artery. Light green arrows, height of the balcony; light blue arrow, mucosa thickness.

The radiographic assessments were carried out in the coronal view using the i-Dixel 2.0 software (J. Morita Corporation, Kyoto, Japan) by a well-trained examiner (KAAA). The floor of the nose was used as the horizontal reference plane, and a line crossing the anterior nasal spine and the nasal septum as a vertical plane was applied for the radiological evaluations (*X*-axis; Figure 1A–C) [10,11]. All measurements were performed at the level of the mini-implant inserted.

### 2.5. Radiographic Evaluations

The following landmarks were identified in the CBCT in the coronal view (Figure 1):

(i) at time 0 (t-0), the floor of the nose (*X*-axis), the center of the bony crest (C), the base of the sinus floor (F), the alveolar-antral artery (AAA), and the palatal nasal recess (PNR).
(ii) at time 1 week (t-1w), the upper (UM) and lower margin (LM) of the antrostomy, the uppermost extension of the hard tissue within the elevated space at the medial, intermediate, and lateral aspects.
(iii) at time 9 months (t-9m), the uppermost extension of the hard tissue within the elevated space at the medial (M), intermediate (I), and lateral (L) aspects.

**Figure 2:**
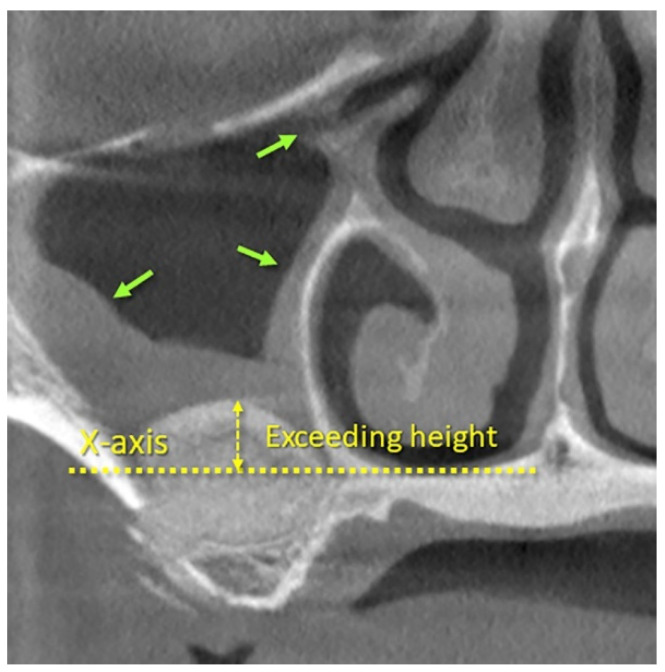
CBCT image illustrating the radiographic aspect of the sinus after 1 week from surgery. Note that the post-surgical edema of the sinus mucosa extended along the sinus walls, involving ostium and infundibulum (green arrows). The yellow arrow indicates the exceeding height that represents the height of the hard tissue above the *X*-axis.

The following parameters were assessed at the level of the implant site: mucosa thickness in the intermediate aspect (MT), bone crest height (distance CF), height of interest (distance XF), that is, the distance between the base of the sinus (F) and the *X*-axis, the most coronal location of the hard tissue (gain) at the three aspects, medial (MF), intermediate (IF), and lateral (LF), exceeding height above *x*-axis (EH; Figure 2), the area of the elevated space, enclosed by the sinus bone walls and the coronal contour of the hard tissue (Area), the area enclosed by the sinus bone walls and the X-axes (X-area), width of interest (XW; i.e., distance between the medial and lateral sinus bone walls on the *X*-axis), angle of the palato-nasal recess (PNR angle), angle between the buccal and palatal bone walls at the sinus floor (sinus floor angle; SFA), and the width of the lateral sinus wall evaluated at 3 mm and 9 mm from the sinus floor. The height available for implant insertion was calculated as CF + IF.

### 2.6. Data Analysis

The radiographic assessments were performed twice by a well-trained assessor (K.A.A.A.) with an intra-examiner coefficient k > 0.8 for all variables and means were calculated for the two measurements. Mean values and standard deviations (SD) were subsequently calculated for each variable.

The Spearman two-tailed correlation coefficient was applied to measure the strength of the correlation between two variables using GraphPad Prism 9.1.1 (GraphPad Software, LLC, San Diego, CA, USA). For interpretation of the correlation coefficients, the strength was expressed according to Dancey and Reidy [32,33]. The correlation coefficient, *p*-values, and 95% confidence interval were reported. A *p*-value < 0.05 was considered statistically significant. The main endpoints were the height gain in the intermediate region (IF) and the changes of the mucosa thickness between 1w and t0 (MT Δ1w–t0).

The data were stratified regarding the biomaterials and correlations were analyzed with height gain, and mucosa thickness changes. The volume of the biomaterial and the length of the antrostomy, both assessed clinically, were correlated with the various parameters analyzed.

## 3. Results

The previous RCTs that included the same sample of patients presented as exclusion criteria patients who were heavy smokers. However, in that population, none of the patients were smokers. Seventy-eight sinuses were evaluated, 58 elevated using porcine xenograft, and 20 elevated using bovine xenograft. Sixty-eight antrostomies were protected with a collagen membrane, and ten were left unprotected. Sixteen collagen membranes were placed subjacent to the sinus mucosa, six of which aimed to protect small perforations.

### 3.1. Anatomical Parameters and Dimensional Changes Overtime in the Subantral Space

After one week of healing, the sinus floor was elevated by 7.1 ± 2.7 mm, 11.1 ± 2.8 mm, and 8.3 ± 2.6 mm at the medial, intermediate, and lateral aspects, respectively (Table 1). After nine months of healing, the respective values were reduced to 6.7 ± 2.4 mm, 9.4 ± 3.0 mm, and 7.8 ± 2.3 mm. The available height for implant insertion including the alveolar crest (3.2 ± 1.3) was 14.3 ± 3.0 mm after one week, and 12.6 ± 3.0 after nine months. The mean height of the balcony was 3.6 ± 1.3 mm while the mean height of the antrostomy was 5.7 mm.

The mucosa width was 2.7 ± 3.7 mm before surgery. The mean width increased to 6.4 ± 5.5 mm for the post-surgical bleeding and edema, and decreased to 1.7 ± 2.1 mm after nine months.

### 3.2. Correlations with the Height Gain

**Figure 3:**
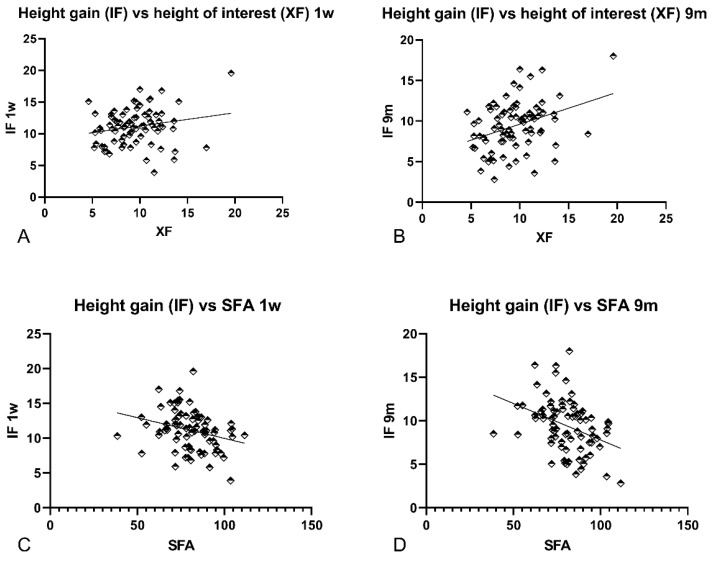
Graphs illustrating correlations between IF and XF at (**A**) 1 week and (**B**) 9 months of healing, and between IF and SFA, at (**C**) 1 week and (**D**) 9 months of healing. IF, distance between the uppermost extension of the hard tissue within the elevated space at the intermediate aspect and F; XF, distance between *X*-axis and sinus floor F; SFA, sinus floor angle.

A weak positive correlation was observed between sinus height of interest (XF) and the height gain of the elevated space after both one week and nine months at the medial (MF), intermediate (IF; Figure 3A,B; Graph XF vs. IF 1w and 9m), and lateral (LF) aspects (Table 2). After nine months, the correlation coefficients were 0.34 (*p*-values 0.003), 0.31 (*p*-values 0.006), and 0.37 (*p*-values 0.0008) at the MF, IF, and LF, respectively.

The height of the elevated space presented a weak correlation or zero with the PNR angle, however, with non-significant *p*-values. SFA showed a weak negative correlation in both periods of observation (Figure 3C,D; SFA vs. IF 1w and 9m), presenting *p*-values < 0.05 at all aspects after nine months.

IF presented a week positive correlation with the balcony height (LM-F) both after one week (r = 0.26; *p*-value 0.023) and nine months (r = 0.27; *p*-value 0.018). The height of the antrostomy (LM-UM) presented weak negative correlations or zero in both periods examined.

The correlation of the elevated space height changed at the intermediate aspect of the sinus between nine months and one week (IF Δ9m–1w) vs. the other parameters evaluated was from weak to zero for both biomaterials (Table 3).

Height gain at the intermediate aspect (IF) and height change between nine months and one week (IF Δ9m–1w) were also correlated to the volume of xenograft used (Table 4) for sinus floor elevation and to the length of the antrostomy (Table 5), as evaluated clinically. The correlations were weak or zero in both analyses excluding a negative moderate correlation for the bovine xenograft (r = −0.44; *p*-values 0.053).

A moderate negative correlation was found between XF and EH at both one week (r = −0.58; *p*-value < 0.001; 95% −0.72 to −0.41) and nine months (r = −0.46; *p*-value < 0.001; 95% −0.62 to −0.26).

The correlation after nine months between the height gain IF and the dimensions of the antrostomies as evaluated clinically (65.3 ± 23.4 mm^2^) was r = −0.20 (*p*-value 0.07; 95% CI −0.41 to 0.026).

### 3.3. Correlations with the Mucosa Thickness after One Week of Healing (MT)

**Figure 4:**
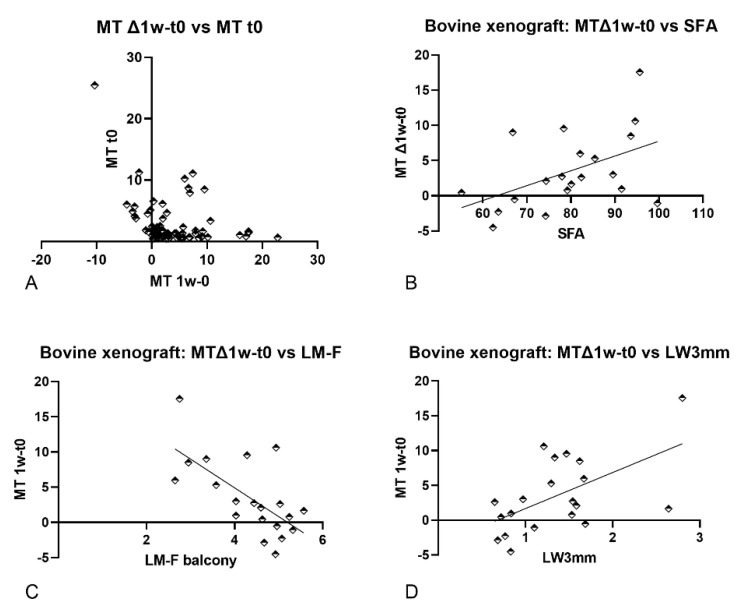
Graphs illustrating the correlations between MT Δ1w–t0 and various parameters. (**A**) Correlation between MT Δ1w–t0 and MT t0 (mucosa thickness baseline). The stratification of the biomaterials showed moderate correlations between MT Δ1w–t0 and (**B**) SFA, (**C**) LM-F (balcony) and (**D**) LW3mm for the bovine xenograft. MT t1w–t0, difference in thickness of the sinus mucosa between t1w and t0; MT t0, mucosa thickness baseline; SFA, sinus floor angle; LM-R, balcony; LW3mm, Lateral bone wall thickness at 3 mm from F.

The mucosa width changes between t0 and t1w ranged between weak and zero correlation vs. all anatomical parameters evaluated (Figure 4; Table 6). A weak negative correlation was also found between the mucosa width at t0 and t1w (r = −0.25; *p*-value 0.025). When the data were stratified regarding the biomaterials, bovine xenograft showed moderate positive correlations vs. SFA (r = 0.50; *p*-value 0.026) and LW3mm (r = 0.47; *p*-value 0.038) and a moderate negative correlation vs. LM-F (balcony; r = −064; *p*-values 0.003).

## 4. Discussion

### 4.1. Anatomical Parameters and Dimensional Changes Overtime in the Subantral Space

The use of the base of the nasal floor (*x*-axis) as the reference plane was adopted for both bi-dimensional [10,11] and 3-dimensional [15] evaluations. The region of interest of the present study (i.e., the region where implants are inserted) was delimited by the sinus walls and the *x*-axis. The mean distance between the *X*-axis and the sinus floor was 9.5 mm. This distance approximately corresponded to the position of the palate-nasal recess (PNR). A classification based on the sinus depth has recently been proposed. Three classes were included, based on the location of the sinus floor with respect to the hard palate [9]: (I) the sinus floor located above the hard palate; (II) 0–6 mm below; and (III) >6 mm. In the present study, no class I was detected, while eight sinuses were classified as class II, one 4.6 mm, and seven between 5 and 6 mm in height. The remaining sinus floors were classified as class III.

The mean width of the sinus as measured on the *X*-axis was 15.5 mm. This distance approximately corresponds to the distance to the PNR from the lateral sinus bone wall. After one week of healing, the level of the hard tissue was 11.1 mm above the sinus floor at the intermediate aspect, and 7.1 mm and 8.3 mm at the medial and lateral aspects, respectively, providing a dome aspect to the elevated space. The mean height of the bone crest was 3.2 mm so that the total height available for implant insertion at the intermediate aspect was 14.3 mm, and reduced to 12.6 mm after nine months of healing, yielding a vertical gain of 9.4 mm. A similar gain (8.5–8.7 mm) was also reported in a randomized controlled trial that compared the healing after six months at sinuses elevated using antrostomies of different heights [16]. In a retrospective study, the influence of the height of the antrostomies was also evaluated. An increased height of the sinus floor of 9.5 mm at the smaller and 10.4 mm at the higher antrostomies was obtained [17] without presenting a statistically significant difference.

In the present study, all antrostomies were prepared using a sonic instrument. This instrument has been shown to be effective in oral surgery and sinus floor elevation as evaluated clinically [28,34,35,36,37,38] and histologically [39,40,41,42].

The mean sinus mucosa thickness before surgery was 2.7 mm. The width increased to 6.4 mm after one week of healing for the edema interposed between the sinus mucosa and the biomaterial after sinus floor elevation. Given that it was not possible on the CBCT to discriminate between edema and sinus mucosa, the term of “virtual mucosa thickness” has been used to describe the increased dimensions as evaluated on the tomography [28]. After nine months, the sinus mucosa width decreased to 1.7 mm.

The edema after surgery has been described in clinical study both after transcrestal [21] and lateral sinus floor elevation [21,22,43].

### 4.2. Correlations with the Height Gain

Weak positive correlations were found between the sinus height of interest (XF) and the height gain of the elevated space in all aspects (MF, medial; IF, intermediate; LF, lateral) and periods of evaluation (one week and nine months). The parameter XF delimits the coronal border of the region of interest below the *X*-axis and a positive correlation indicates that the higher the XF, the higher the height gain. It has to be considered that the parameter XF also roughly identifies the location of the PNR. An acute PNR angle (<90°) increases the difficulties of detaching the sinus mucosa from the bone and it is considered a risk factor for perforations [23]. In the present study, however, XF was 9.5 mm while the coronal extension of the xenograft at the medial aspect (MF) was 7.1 mm after one week, meaning that the PNR was not reached by the xenograft during the sinus mucosa elevation. From a clinical point of view, the PNR is an important reference for clinicians, especially in sinuses with a reduced XF (height of interest). In such cases, it might be necessary to elevate the sinus mucosa above the *X*-axis, and involving the PNR might in the elevation procedures. Indeed, a moderate negative correlation was found between XF and HE, meaning that the more reduced the XF, the higher the exceeding height above the *X*-axis, obviously aiming to obtain a sufficient gain height of the elevated space for implant insertion.

The SFA presented a weak negative correlation in most aspects, meaning that the more acute the angle, the higher the height gain, due to the reduced dimension presented by an acute SFA.

The balcony also slightly influenced the height gain, presenting a positive weak correlation. In a RCT included in the present study, ten patients in the test group had a balcony height of 1.1 ± 0.9 mm while in the control group, the balcony was 3.5 ± 0.6 mm [10]. In the intermediate aspect, a higher height gain was observed in the control group compared to the test group, showing an effect on the dimension of the elevated space after both one week and nine months. This was also corroborated by the histological evaluation on mini-implants inserted in the same series of elevated sinuses after six months from sinus floor elevation and retrieved three months afterward. A bone-to-implant contact percentage of 48.5% and 40.9% was found in the test and control groups, respectively [14]. This confirmed the importance of leaving bone walls at the base of the sinus also at the lateral aspects, providing an important source for new bone formation from the balcony [44,45,46,47,48,49,50].

The height of the antrostomy presented a weak negative or no correlation with the height gain at all aspects. Correspondingly, the area of the antrostomies, evaluated clinically, presented a negative weak correlation vs. IF at nine months. This correlation, even though weak, might be related to the loss of biomaterial through the antrostomy that might be higher at larger compared to smaller antrostomies [20]. In the present study, all antrostomies had limited dimensions in height, ranging between 4 and 8 mm. Including antrostomies of larger dimensions might yield stronger correlation.

In the present retrospective article, twenty of the patients included were originally recruited in n RCT in which the antrostomies were prepared with a height of either 4 mm or 8 mm [11]. This study showed higher gain at the small compared to large antrostomies, even though the difference was not statistically significant. In a subsequent study from the same sample of patients, the histological healing of mini-implants inserted after six months of healing and retrieved after three months was evaluated [51]. A similar amount of osseointegration was found in both the large and small antrostomies, meaning that a height of antrostomies between 4 and 8 mm also did not influence the osseointegration of implants. Likewise, no difference in new bone content was found in an experiment in rabbits in which antrostomies of different dimensions were prepared [52].

The biomaterial was evaluated as a whole sample for both the volume of biomaterial used and length of the antrostomy. Weak or no correlations were found. The sample was stratified according to the xenograft type, yielding similar outcomes. Only the height changes between nine months and one week (IF 9m–1w) presented a moderate correlation (*p*-value 0.053).

### 4.3. Correlations with the Mucosa Thickness Changes after One Week of Healing (MT Δ1w–t0)

The increased dimensions of the “virtual mucosa” [28], and consequently of the post-surgical edema, presented a weak or no correlation with the parameters evaluated. When a stratification was performed according to the biomaterials at the porcine xenograft group, weak or no correlations were found. However, at the bovine xenograft, a positive moderate correlation was observed for SFA (0.50; *p*-value 0.026) and LW3mm (0.47; *p*-values 0.038), and a negative moderate correlation was found for the LM-F (balcony). This outcome might be related to the small sample included in the bovine compared to the porcine xenografts [33], even though the *p*-values were <0.05. However, the different dimensions and characteristics of the two biomaterials might have played an effect. The bovine xenograft had granules of 1–2 mm of dimensions, while the porcine xenograft had smaller granules (0.250–1 mm). The larger dimensions of the granules used in small antrostomies might have increased the trauma on the regions close to the base of the sinus presenting acute SFA, thick LW3mm, and a low balcony.

In the present study, the sinus mucosa width was measured vertically in an intermediate position above the hard tissue. However, it has been shown that the edema is not limited to the region overlying the elevated space, but also spreads along the walls of the sinus, involving ostium and infundibulum. Out of seventy-two sinuses evaluated one week after sinus floor elevation, 14 were found devoid of infundibulum patency (Figure 2). However, after nine months, only one infundibulum was still out of patency [28].

In one of the RCT included in the present retrospective study [14], a collagen membrane was placed subjacent to the sinus mucosa at the test sites with the aim to evaluate the effect on height changes of the elevated space. The presence of the collagen membrane resulted in a lower loss of height and thickening of the sinus mucosa compared to the control sites. However, the differences were not statistically significant.

### 4.4. Limitations of the Study

As limitations of the present study, the retrospective design together with the 2-dimensional analysis performed should be included. A 3-dimensional analysis would have provided volumetric information [53,54]. The number and heterogeneity of the variables analyzed and the use of two different types of filler materials and membranes are other limitations. In fact, higher reductions in the height of the elevated space were reported in sites at which a porcine collagenated material was used (range 1.4–3 mm) [10,11,14] compared to those filled with a bovine xenograft (0.6–0.8 mm) [13]. The use of a collagen membrane subjacent to the sinus mucosa or on the antrostomy did not result into major differences between height changes of sinus mucosa thickening [10,11,13,14].

Another limitation of the present study is represented by the tomographic evaluation that could not discriminate between edema and sinus mucosa. For this reason, the term of “virtual mucosa thickness” was introduced [28]. However, the contribution of the sinus mucosa in increasing this “virtual mucosa thickness” is limited. The histological changes of the thickness of the mucosa after sinus augmentation were studied in rabbits [47,55]. The pristine sinus mucosa presented a thickness of about 70–80 µm in both studies. In an experiment [47], the subantral space was elevated with a collagenated porcine xenograft. Between 2 and 8 weeks, the sinus mucosa increased the dimensions by about 100 µm, mainly due to gland proliferations. In another experiment [55], either deproteinized bovine bone mineral or autogenous bone were used as fillers. After seven and 40 days of healing, several regions of the sinus mucosa were found presenting a reduced mucosa thickness (<40 µm), especially in the DBBM group.

Despite the limitations, the similar inclusion and exclusion criteria applied and the similar periods of examination as well as the number of sinuses examined in the present study controlled a possible bias.

More studies should be performed using different biomaterials and applying antrostomies of different dimensions to evaluate the post-surgical submucosal edema, aiming to limit its extension toward the ostium and avoid loss of patency.

## 5. Conclusions

Various parameters might affect height gain and sinus mucosa thickening after sinus floor elevation. The height of interest, the balcony, and the sinus floor angle showed significant correlations with height gain. The initial thickness of the mucosa and the biomaterial used influenced the post-surgical edema.

## Figures and Tables

**Table 1:** Anatomical parameters and dimensional changes as assessed on the CBCTs taken at the baseline (t0), and 1 week (t1w) and 9 months (t9m) after sinus floor elevation. Data in millimeters excluding * expressed in degrees; *n* = 78.

		t0	t1w	t9m	t1w–t0	t9m–t1w	t9m–t0
MT	Sinus mucosa thickness	2.7 ± 3.7	6.4 ± 5.5	1.7 ± 2.1	3.7 ± 5.3	−4.7 ± 5.8	−1.0 ± 4.2
CF	Bone crest height	3.2 ± 1.3					
XF	Distance from *x*-axis and sinus floor (height of interest)	9.5 ± 2.7					
MF	Height gain from F at the medial aspect		7.1 ± 2.7	6.7 ± 2.4		−0.4 ± 1.6	
IF	Height gain from F at the intermediate aspect		11.1 ± 2.8	9.4 ± 3.0		−1.7 ± 2.0	
LF	Height gain from F at the lateral aspect		8.3 ± 2.6	7.8 ± 2.3		−0.5 ± 1.4	
EH	Exceeding height at *X*-axis		1.6 ± 3.5	−0.1 ± 3.3		1.7 ± 2.0	
X-area	Area enclosed by the sinus bone walls and the *X*-axis (area of interest)	99.3 ± 41.6					
Area	Area of the elevated space		100.7 ± 32.0	80.6 ± 33.2		−20.1 ± 21.5	
XW	Distance between the medial and lateral sinus bone walls on the *X*-axis						
PNR *	Palato-nasal recess angle	129.1 ± 22.9					
SFA *	Sinus floor angle	80.6 ± 13.3					
LW3	Lateral bone wall thickness at 3 mm from F	1.3 ± 0.6					
LW9	Lateral bone wall thickness at 9 mm from F	1.2 ± 0.5					
LM-F	Balcony; distance between the lower margin of the antrostomy (LM) and the sinus floor (F)		3.6 ± 1.3				
LM-UM	Antrostomy height		5.7 ± 1.1				
UM-F	Distance between the upper margin of the antrostomy (UM) and the sinus floor (F)		9.3 ± 1.6				
AAA	Alveolar-antral artery distance from C	16.9 ± 3.1					
CF + IF	Available height for implant insertion		14.3 ± 3.0	12.6 ± 3.0		−1.7 ± 2.0	

**Table 2:** Correlations with height gain at the medial (MF), intermediate (IF), and lateral (LF) aspects of the sinus after 1 week (1w) and 9 months (9m) of healing. Data not stratified based on biomaterial type; *n* = 78.

		MF 1w	IF 1w	LF 1w	MF 9m	IF 9m	LF 9m
XF; Distance from *x*-axis and sinus floor (height of interest)	r	0.28	0.17	0.19	0.34	0.31	0.37
	*p*-value	0.015	0.144	0.095	0.003	0.006	0.0008
	95% CI	0.049 to 0.47	−0.06 to 0.38	−0.04 to 0.40	0.12 to 0.52	0.09 to 0.50	0.16 to 0.56
XW; Distance between medial and lateral sinus bone walls on *X*-axis (width of interest)	r	0.02	−0.02	−0.10	0.05	0.09	0.08
	*p*-value	0.845	0.829	0.368	0.661	0.437	0.486
	95% CI	−0.21 to 0.25	−0.25 to 0.21	−0.33 to 0.13	−0.18 to 0.28	−0.14 to 0.31	−0.15 to 0.30
PNR angle; Palato-nasal recess angle	r	−0.19	−0.11	0.0003	−0.20	−0.20	−0.05
	*p*-value	0.105	0.35	0.998	0.082	0.076	0.684
	95% CI	−0.40 to 0.05	−0.33 to 0.13	−0.23 to 0.23	−0.41 to 0.03	−0.41 to 0.03	−0.27 to 0.18
SFA; Sinus floor angle	r	−0.18	−0.33	−0.28	−0.29	−0.39	−0.34
	*p*-value	0.123	0.004	0.012	0.01	0.0004	0.002
	95% CI	−0.39 to 0.055	−0.52 to −0.10	−0.48 to −0.06	−0.49 to −0.07	−0.57 to −0.18	−0.53 to −0.12
LM-F: balcony	r	0.15	0.26	0.23	0.11	0.27	0.18
	*p*-value	0.194	0.023	0.044	0.335	0.018	0.118
	95% CI	−0.08 to 0.37	0.03 to 0.46	0.0002 to 0.44	−0.12 to 0.33	0.04 to 0.47	−0.05 to 0.39
LM-UM; height of the antrostomy	r	−0.23	−0.08	−0.09	−0.22	−0.20	0.044
	*p*-value	0.044	0.5	0.411	0.059	0.079	0.7
	95% CI	−0.44 to 0.0003	−0.30 to 0.15	−0.32 to 0.14	−0.42 to 0.01	−0.41 to 0.03	−0.19 to 0.27

Spearman correlation coefficient (r), two-tailed *p*-value, 95% confidence interval (CI).

**Table 3:** Correlations with gain height changes at the intermediate aspect (IF) of the sinus between 9 months and 1 week (IF Δ9m–1w). Data are presented stratified for both biomaterials.

		Both Biomaterials (*n* = 78)	Porcine Xenograft (*n* = 58)	Bovine Xenograft (*n* = 20)
XF; Distance from *x*-axis and sinus floor (height of interest)	r	0.25	0.25	0.04
	*p*-value	0.03	0.061	0.869
	95% CI	0.02 to 0.45	−0.02 to 0.48	−0.42 to 0.48
XW; Distance between medial and lateral sinus bone walls on the *X*-axis (width of interest)	r	0.23	0.21	0.17
	*p*-value	0.045	0.109	0.484
	95% CI	−0.0008 to 0.43	−0.06 to 0.45	−0.31 to 0.58
PNR angle; Palato-nasal recess angle	r	−0.19	−0.096	−0.18
	*p*-value	0.094	0.4737	0.448
	95% CI	−0.40 to 0.04	−0.35 to 0.17	−0.59 to 0.30
SFA; Sinus floor angle	r	−0.04	−0.06	−0.05
	*p*-value	0.702	0.676	0.823
	95% CI	−0.27 to 0.19	−0.32 to 0.21	−0.50 to 0.41
LM-UM; height of the antrostomy	r	−0.12	−0.12	0.19
	*p*-value	0.278	0.369	0.411
	95% CI	−0.34 to 0.11	−0.37 to 0.15	−0.28 to 0.60

Spearman correlation coefficient (r), two-tailed *p*-value, 95% confidence interval (CI).

**Table 4:** Correlations between xenograft volume used for elevation and height gain at the intermediate aspect (IF) after 1 week (1w) and 9 months (9m), and height gain changes between 9 months and 1-week (IF Δ9m–1w). Evaluations based on clinical data on xenograft volume used. Data are presented stratified for both biomaterials.

		Both Biomaterials (*n* = 78)	Porcine Xenograft (*n* = 58)	Bovine Xenograft (*n* = 20)
IF 1w; Height gain in the intermediate region after 1 week	r	0.19	0.05	0.2
	*p*-value	0.092	0.711	0.396
	95% CI	−0.04 to 0.40	−0.22 to 0.31	−0.28 to 0.60
IF 9m; Height gain in the intermediate region after 9 months	r	0.16	−0.17	0.19
	*p*-value	0.163	0.205	0.432
	95% CI	−0.07 to 0.37	−0.42 to 0.10	−0.29 to 0.59
IF Δ9m−1w; Height changes between 9 months and 1 week	r	−0.02	−0.26	0.18
	*p*-value	0.847	0.0498	0.446
	95% CI	−0.25 to 0.21	−0.49 to 0.007	−0.30 to 0.59

Spearman correlation coefficient (r), two-tailed *p*-value, 95% confidence interval (CI).

**Table 5:** Correlations between length of the antrostomy and height gain (IF) after 1 week (1w) and 9 months (9m), and height gain changes between 9 months and 1-week periods. Evaluations based on clinical data on the length of the antrostomy. Data are presented stratified for both biomaterials.

		Both Biomaterials (*n* = 78)	Porcine Xenograft (*n* = 58)	Bovine Xenograft (*n* = 20)
IF 1w; Height gain in the intermediate region after 1 week	r	−0.15	−0.26	0.21
	*p*-value	0.202	0.0504	0.367
	95% CI	−0.36 to 0.09	−0.49 to 0.008	−0.27 to 0.61
IF 9m; Height gain in the intermediate region after 9 months	r	−0.16	−0.18	0.1
	*p*-value	0.17	0.1712	0.663
	95% CI	−0.37 to 0.08	−0.43 to 0.09	−0.37 to 0.53
IF 9m−1w; Height gain changes between 9 months and 1 week	r	−0.008	0.14	−0.44
	*p*-value	0.944	0.289	0.053
	95% CI	−0.24 to 0.22	−0.13 to 0.39	−0.74 to 0.02

Spearman correlation coefficient (r), two-tailed *p*-value, 95% confidence interval (CI).

**Table 6:** Correlations with mucosa thickness changes (MT Δ1w-0) between 1 week (1w) of healing and baseline (t0). Data for both biomaterials and stratified for type; *n* = 78.

		Both Biomaterials (*n* = 78)	Gen-Os (*n* = 58)	Cerabone (*n* = 20)
XF; Distance from *x*-axis and sinus floor (height of interest)	r	0.09	0.18	−0.16
	*p*-value	0.448	0.168	0.489
	95% CI	−0.14 to 0.31	−0.09 to 0.43	−0.58 to 0.31
XW; Distance between medial and lateral sinus bone walls on the *X*-axis (width of interest)	r	0.21	0.22	0.12
	*p*-value	0.063	0.099	0.627
	95% CI	−0.02 to 0.42	−0.05 to 0.46	−0.36 to 0.54
PNR angle; Palato-nasal recess angle	r	−0.02	−0.10	0.18
	*p*-value	0.891	0.472	0.447
	95% CI	−0.24 to 0.21	−0.35 to 0.17	−0.30 to 0.59
SFA; Sinus floor angle	r	0.20	0.11	0.50
	*p*-value	0.076	0.416	0.026
	95% CI	−0.03 to 0.41	−0.16 to 0.36	0.056 to 0.78
LM-F: balcony	r	−0.18	0.02	−0.64
	*p*-value	0.120	0.884	0.003
	95% CI	−0.39 to 0.05	−0.25 to 0.28	−0.85 to −0.26
LM-UM; height of the antrostomy	r	0.22	0.23	0.28
	*p*-value	0.058	0.076	0.225
	95% CI	−0.014 to 0.42	−0.03 to 0.47	−0.19 to 0.65
MT t0; mucosa width at t0	r	−0.25	−0.25	−0.23
	*p*-value	0.025	0.055	0.339
	95% CI	−0.46 to −0.026	−0.49 to 0.01	−0.62 to 0.25
LW3mm; lateral wall thickness at 3 mm from sinus floor F	r	0.18	0.07	0.47
	*p*-value	0.106	0.595	0.038
	95% CI	−0.05 to 0.40	−0.20 to 0.33	0.02 to 0.76
LW9mm; lateral wall thickness at 9 mm from sinus floor F	r	−0.06	−0.07	−0.03
	*p*-value	0.577	0.590	0.9072
	95% CI	−0.29 to 0.17	−0.33 to 0.20	−0.48 to 0.43

Spearman correlation coefficient (r), two-tailed *p*-value, 95% confidence interval (CI).

## Data Availability

The data are available on reasonable request.
